# The expression of histone deacetylase HDAC1 correlates with the progression and prognosis of gastrointestinal malignancy

**DOI:** 10.18632/oncotarget.16843

**Published:** 2017-04-05

**Authors:** Lin-Lin Cao, Zhihong Yue, Lianhua Liu, Lin Pei, Yue Yin, Li Qin, Jie Zhao, Huixin Liu, Hui Wang, Mei Jia

**Affiliations:** ^1^ Department of Clinical Laboratory, Peking University People's Hospital, Beijing 100044, People's Republic of China; ^2^ Research Office, Peking University People's Hospital, Beijing 100044, People's Republic of China

**Keywords:** HDAC1, meta-analysis, gastrointestinal malignancy, clinical feature, overall survival

## Abstract

Gastrointestinal malignancy is a severe public health threat worldwide, and survival for most types of gastrointestinal cancer is very poor. Therefore, finding better cancer biomarkers to diagnose gastrointestinal malignancy and predict patient survival is essential. HDAC1 has been reported to be closely associated with several types of cancer, but the precise role of HDAC1 in gastrointestinal cancer is not clear. Recently, quite a few studies have investigated the correlation between HDAC1 expression and clinical features or prognosis in multiple types of gastrointestinal malignancies, but the results were inconsistent. In this study, we systematically reviewed the association between HDAC1 and gastrointestinal malignancy using meta-analysis methods, and 28 eligible studies were analyzed. We found that the expression level of HDAC1 in gastrointestinal malignancies, especially in colorectal cancer (OR = 10.84, 95% CI = 5.33–22.07, P< 0.00001), was higher than that in noncancerous tissue, and HDAC1 expression was closely associated with some clinical features of gastrointestinal cancer patients, such as tumor stage (OR = 1.62, 95% CI = 1.28–2.05, P < 0.0001) and tumor grade (OR = 1.75, 95% CI = 1.03–2.95, P = 0.04). In addition, we also found that patients with low HDAC1 expression showed better overall survival than those with high HDAC1 expression in gastrointestinal malignancy, especially in gastric cancer (HR = 1.88, 95% CI = 1.14–3.12, P = 0.01). Our results strongly suggest that HDAC1 may serve as a good diagnostic and prognostic marker for gastrointestinal malignancy.

## INTRODUCTION

Gastrointestinal malignancies present an ever-increasing global public health threat, including several types of cancer, such as esophageal, gastric, liver, pancreatic and colorectal cancer. These malignancies have a high mortality rate, especially in less developed regions of the world [1]. The overall survival (OS) rates for gastrointestinal malignancy remain low despite advances in early diagnosis and clinical treatments over the last several decades [2]. Therefore, the identification of new biomarkers to screen out high-risk patients and predict gastrointestinal cancer prognosis is urgent.

An overwhelming number of studies have proven that Histone Deacetylase 1 (HDAC1) is tightly correlated with cancer. For example, HDAC1 has been demonstrated to be overexpressed in many cancers, such as in breast, lung and renal cell cancer, as well as in classical Hodgkin's lymphoma [3–6]. In addition, HDAC1 overexpression is often associated with poor prognosis in breast and lung cancer [3, 4]. Moreover, HDAC1 silencing by siRNA results in cell cycle arrest, cell growth inhibition, and induction of apoptosis in breast and colon cancer cells [7, 8], while HDAC1 overexpression leads to an increase in cell proliferation in prostate cancer cells [9], indicating that HDAC1 stimulates cancer cell growth. Taken together, these findings suggest that HDAC1 may be a good diagnostic and prognostic marker for some types of cancer. However, the role of HDAC1 in the progression and prognosis of gastrointestinal malignancy is largely unknown.

In this study, we performed a systematic review and a meta-analysis to evaluate the correlation of HDAC1 expression with several types of gastrointestinal malignancy. We found that gastrointestinal cancer tissue showed higher HDAC1 expression than normal tissue, and HDAC1 expression was associated with several clinical features of gastrointestinal malignancy. Additionally, HDAC1 expression was negatively correlated with the OS rate of patients with gastrointestinal malignancies, especially gastric cancer. Overall, this study is the first to systematically review the critical role of HDAC1 in the progression and prognosis of gastrointestinal malignancy.

## RESULTS

### Study selection and characteristics of the included studies

**Figure 1:**
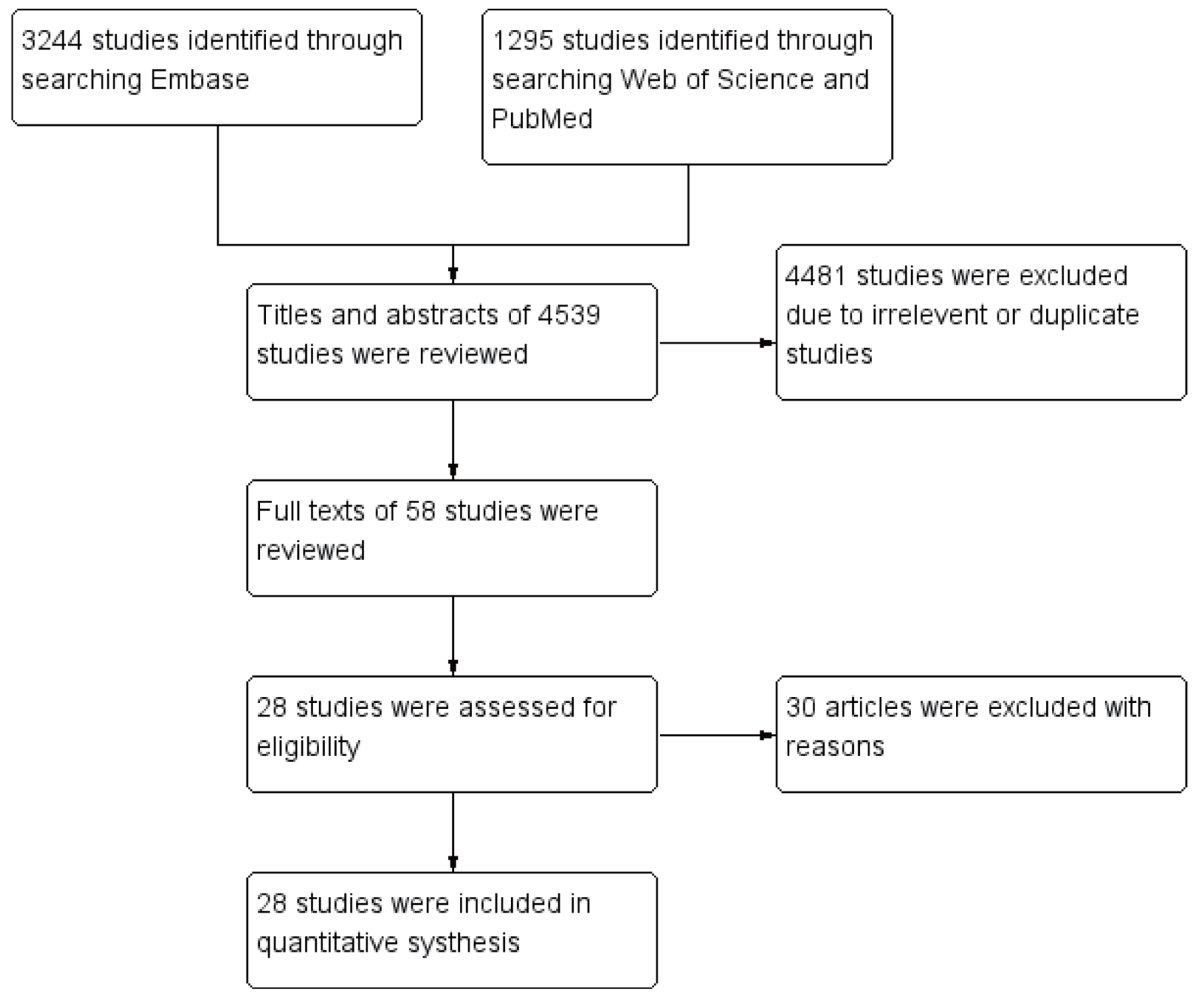
Methodological flow chart of study selection

A total of 4539 papers were retrieved using the search strategy (Figure 1). After the paper titles and abstracts were checked, 4481 studies were excluded because of their irrelevance and duplication. Then, the remaining 58 articles were viewed in their entirety. Among the 58 articles, 30 were excluded due to the following reasons: (1) western blot was used to determine HDAC1 expression; (2) the studies only focused on animal models or cell lines; or (3) relative data could not be extracted. Finally, 28 studies [8, 10–36] matched the criteria for this analysis, including 1 study concerning colorectal, gastric and esophageal cancer, 9 studies concerning only colorectal cancer, 5 studies concerning only gastric cancer, 1 study concerning only esophageal cancer, 5 studies concerning only liver cancer and 7 studies concerning only pancreatic cancer.

All characteristics of the 28 studies are listed in Table 1. Among the studies, sixteen originated from Asia, eleven from Europe and one from America. A total of 2368 cases were enrolled. An immunohistochemistry assay was used in 22 studies, whereas a real-time polymerase chain reaction (RT-PCR) assay was used in six studies. The Newcastle-Ottawa Scale (NOS) was used to assess the methodological quality of the included studies, and the results showed that all the studies had high-quality (Table 2).

**Table 1:** Main characteristics and results of the included studies

Study	Year	Country	Sample size	Age	Detection method	Cut-off value	Histology	Stage	Follow-up period (month)	Outcome
Thangaraju	2009	USA	18	NR	RT-PCR	NR	CRC	NR	NR	NR
Huang	2005	Singapore	45	NR	IHC	Score ≥ 2	CRC	NR	NR	NR
Özdağ	2006	UK	20	NR	RT-PCR	NR	CRC	NR	NR	NR
Weichert-1	2008	Germany	140	65.0 (median)	IHC	IRS > 6	CRC	I-IV	64 months (median)	OS
Benard	2015	Netherlands	254	NR	IHC	>Median	CRC	I-III	103.2 months (mean)	OS, DSS, DRFS, LRRFS
Mimori	2005	Japan	61	NR	RT-PCR	T/N ratio >1.65	CRC	NR	NR	OS
Ishihama	2007	Japan	64	NR	IHC	SI >10.7	CRC	I-IV	72 months	OS
Higashijima	2011	Japan	74	67.3 (mean)	IHC	>10%	CRC	I-IV	NR	OS, DFS
Liu	2010	China	94	NR	IHC	SI ≥ 23	CRC	I-IV	60 months	OS
Nakagawa	2007	Japan	20	NR	IHC	Score ≥ 3	CRC, EC, GC	NR	NR	NR
Giaginis	2015	Greece	70	66.77 ± 8.94 (mean)	IHC	Score ≥ 3	PC	I-IV	21 months	OS
Ouaïssi	2008	France	11	NR	RT-PCR	NR	PC	I-IV	NR	NR
Lehmann	2009	Germany	81	66.0 (median)	IHC	IRS > 6	PC	I-IV	NR	OS
Wang	2009	China	54	57.6 (mean)	IHC	>Median	PC	I-IV	NR	OS
Miyake	2008	Japan	39	64.4 (mean)	IHC	≥10%	PC	I-IV	NR	OS
Gao	2010	China	30	59.36 (mean)	IHC	≥Mean	PC	I-III	NR	NR
Ouaïssi	2014	France	11	NR	RT-PCR	NR	PC	NR	16 months (mean)	OS, DFS
Langer	2010	Germany	126	NR	IHC	SI > 6	EC	I-IV	NR	NR
Ler	2015	Singapore	156	58.16 (mean)	IHC	Score ≥ 1	LC	I-IV	120 months	NR
Morine	2012	Japan	35	68 (mean)	IHC	≥10%	LC	I-IV	27.0 months (mean)	OS, DFS
Quint	2011	Germany	170	61.7 ± 11.2 (mean)	IHC	>Median	LC	I-IV	NR	OS
Wu	2010	China	43	NR	IHC	IRS > 6	LC	NR	NR	RFS
Rikimaru	2007	Japan	47	65.3 (mean)	IHC	>Mean	LC	I-IV	NR	OS
Weichert-2	2008	Germany	293	NR	IHC	IRS > 6	GC	I-IV	NR	OS
Yu	2015	China	80	54.4 (mean)	IHC	Mean density ≥ 1.205	GC	I-IV	NR	OS
Mutze	2010	Germany	127	59.9 (mean)	IHC	SI > 6	GC	I-IV	52.8 months (median)	OS
Gao	2012	China	65	65.0 (mean)	IHC	SI > 3	GC	I-IV	NR	NR
Sudo	2011	Japan	140	67.1 (mean)	RT-PCR	≥Mean	GC	I-IV	NR	OS

NR, no reported; IHC, immunohistochemistry; RT-PCR, real-time polymerase chain reaction; IRS, immunoreactivity scoring; SI, staining index; T/N, tumour/normal; CRC, colorectal cancer; EC, esophagus cancer; GC, gastric cancer; PC, pancreatic cancer; LC, liver cancer; OS, overall survival; DFS, disease-free survival; RFS, recurrence-free survival; DRFS, distant recurrence-free survival; LRRFS, loco-regional recurrence-free survival; DSS, disease-specific survival.

**Table 2:** Newcastle-Ottawa Scale for each included study

Study	Selection	Comparability	Exposure	Total quality score
Thangaraju 2009	3	2	3	8
Huang 2005	4	2	3	9
Özdağ 2006	3	0	3	6
Benard 2015	4	0	3	7
Mimori 2005	3	2	3	8
Ishihama 2007	3	2	3	8
Higashijima 2011	3	2	3	8
Nakagawa 2007	3	2	3	8
Langer 2010	3	2	3	8
Weichert-1 2008	3	0	3	6
Liu 2010	3	0	3	6
Giaginis 2015	3	2	3	8
Lehmann 2009	3	2	3	8
Wang 2009	3	2	3	8
Miyake 2008	3	2	3	8
Ouaïssi 2014	3	2	3	8
Gao 2010	3	0	3	6
Ouaïssi 2008	2	1	3	6
Ler 2015	3	2	3	8
Morine 2012	3	2	3	8
Quint 2011	3	2	3	8
Wu 2010	3	2	3	8
Rikimaru 2007	3	2	2	7
Weichert-2 2008	3	2	3	8
Yu 2015	3	2	3	8
Mutze 2010	4	2	3	9
Gao 2012	3	2	3	8
Sudo 2011	3	2	3	8

### The expression level of HDAC1 in gastrointestinal cancer tissues was higher than that in noncancerous tissues

**Figure 2:**
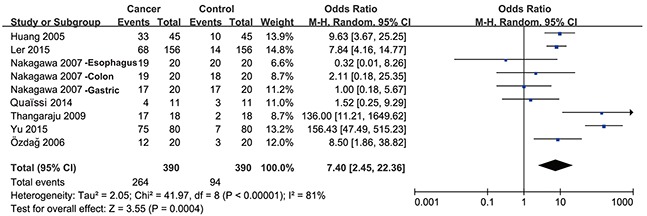
Forest plot of odds ratio (OR) Relative HDAC1 abundance of overall gastrointestinal malignancy in comparison to noncancerous tissues.

Seven studies compared the expression level of HDAC1 between gastrointestinal cancer tissues and noncancerous tissues, including 1 study concerning colorectal, gastric and esophageal cancer, 3 studies concerning only colorectal cancer, 1 study concerning only gastric cancer, 1 study concerning only liver cancer and 1 study concerning only pancreatic cancer. The pooled odds ratio (OR) with 95% confidence interval (CI) including 390 cancer patients is shown in Figure 2 (OR = 7.40, 95% CI = 2.45–22.36, P = 0.0004) and suggests that the HDAC1 expression level in gastrointestinal cancer tissues was higher than that in noncancerous tissues.

**Figure 3:**
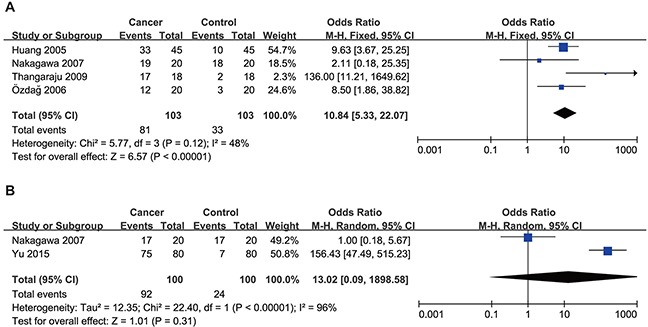
Forest plot of odds ratio (OR) **(A)** Comparison of the expression level of HDAC1 between colorectal cancer tissues and normal tissues. **(B)** Comparison of the expression level of HDAC1 between gastric cancer tissues and normal tissues.

Sub-group analysis was then performed by cancer type. As shown in Figure 3A, the expression level of HDAC1 in colorectal cancer was higher than that in control tissue samples (OR = 10.84, 95% CI = 5.33–22.07, P < 0.00001). There was no significant difference in HDAC1 expression levels between gastric cancer tissues and control tissues (Figure 3B), but only two studies with high heterogeneity (I^2^= 96%) were enrolled. Only one study investigated esophageal, liver or pancreatic cancer separately. Together, these results suggest that the expression level of HDAC1 in gastrointestinal malignancies, especially in colorectal cancer, is higher than that in noncancerous tissues.

### Correlation of HDAC1 expression with the clinical features of gastrointestinal cancer patients

**Figure 4:**
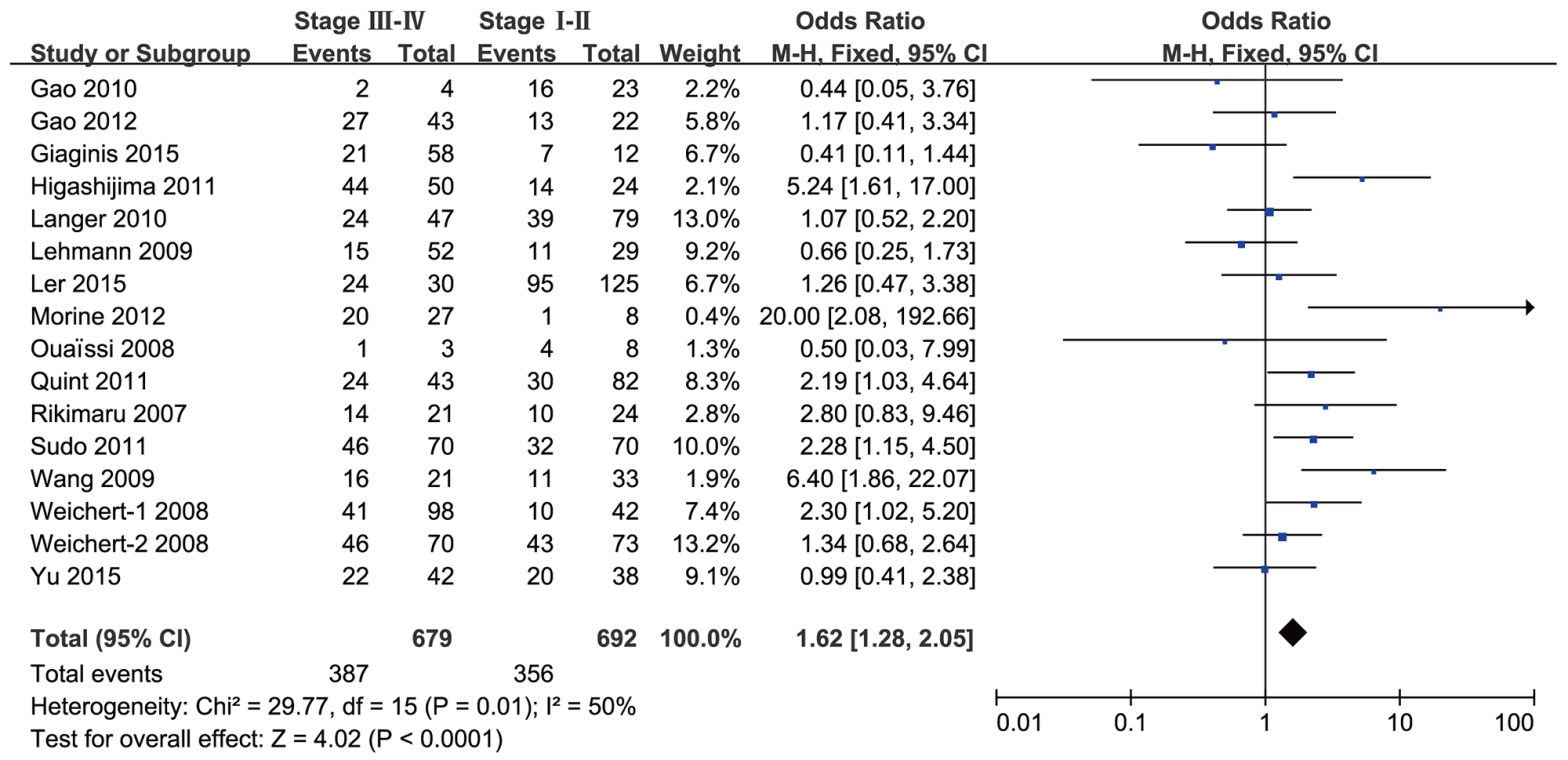
Forest plot of odds ratio (OR) Association between HDAC1 expression and tumor stage in overall gastrointestinal malignancy.

We next analyzed the relationship between HDAC1 expression and the clinical features of gastrointestinal cancer patients. As shown in Figure 4, the HDAC1 expression level was higher in stage III-IV than in stage I-II gastrointestinal cancer patients (OR = 1.62, 95% CI = 1.28–2.05, P < 0.0001). Sub-group analyses were conducted by cancer type as well. As shown in Table 3, the expression level of HDAC1 in stage III-IV was higher than that in stage I-II colorectal cancer (OR = 2.94, 95% CI = 1.50–5.77), gastric cancer (OR = 1.47, 95% CI = 1.00–2.18) and liver cancer (OR = 2.30, 95% CI = 1.38–3.85) but not pancreatic cancer (OR = 1.05, 95% CI = 0.60–1.85) and esophageal cancer (only one study).

**Table 3:** Sub-group analyses were stratified on the basis of histology

	Stage		Grade		Lymph node metastasis		Distant metastasis	
Colorectal cancer	N	OR (95%CI)	N	OR (95%CI)	N	OR (95%CI)	N	OR (95%CI)
	2	2.94 (1.50, 5.77)	2	3.80 (1.46, 9.92)	2	1.50 (0.83, 2.71)	2	3.67 (1.38, 9.79)
Gastric cancer	N	OR(95%CI)	N	OR(95%CI)	N	OR(95%CI)	N	OR(95%CI)
	4	1.47 (1.00, 2.18)	3	1.27 (0.82, 1.96)	4	1.60 (1.07, 2.40)	1	0.95 (0.34, 2.61)
Esophagus cancer	N	OR(95%CI)	N	OR(95%CI)	N	OR(95%CI)	N	OR(95%CI)
	1	1.07 (0.52, 2.20)	1	0.49 (0.24, 1.00)	1	0.64 (0.31, 1.29)	None	None
Liver cancer	N	OR(95%CI)	N	OR(95%CI)	N	OR(95%CI)	N	OR(95%CI)
	4	2.30 (1.38, 3.85)	5	2.50 (1.45, 4.30)	1	6.60 (1.18, 37.03)	None	None
Pancreatic cancer	N	OR(95%CI)	N	OR(95%CI)	N	OR(95%CI)	N	OR(95%CI)
	5	1.05 (0.60, 1.85)	5	1.54 (0.82, 2.90)	4	1.08 (0.62, 1.88)	2	0.80 (0.19, 3.33)

N, study numbers.

**Figure 5:**
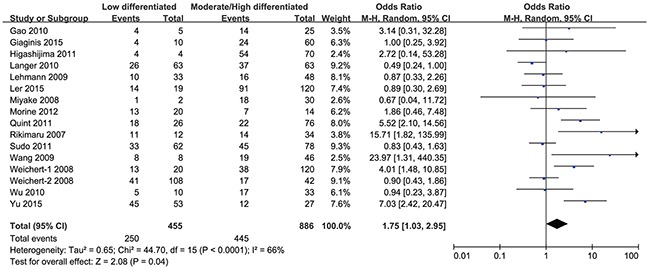
Forest plot of odds ratio (OR) Association between HDAC1 expression and tumor grade in overall gastrointestinal malignancy.

In addition, the HDAC1 expression level was higher in patients with low-differentiated cancer than that expressed in those with moderate/high-differentiated gastrointestinal cancer (OR = 1.75, 95% CI = 1.03–2.95, P = 0.04) (Figure 5). Sub-group analyses (Table 3) showed that the expression level of HDAC1 in the low-differentiated cancer patients was higher than that in the moderate/high-differentiated cancer patients with colorectal cancer (OR = 3.80, 95% CI = 1.46–9.92) and liver cancer (OR = 2.50, 95% CI = 1.45–4.30). However, there was no significant difference in gastric cancer (OR = 1.27, 95% CI = 0.82–1.96) and pancreatic cancer (OR = 1.54, 95% CI = 0.82–2.90). Only one study that investigated the esophageal cancer grade was included.

**Figure 6:**
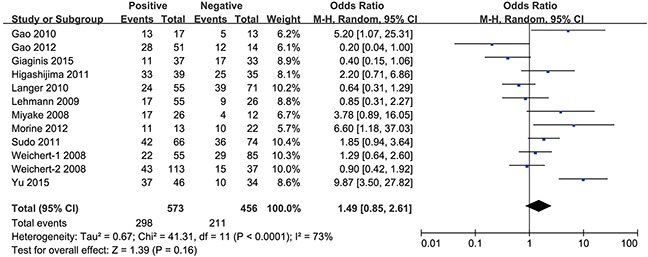
Forest plot of odds ratio (OR) Association between HDAC1 expression and lymph node metastasis in overall gastrointestinal malignancy.

**Figure 7:**
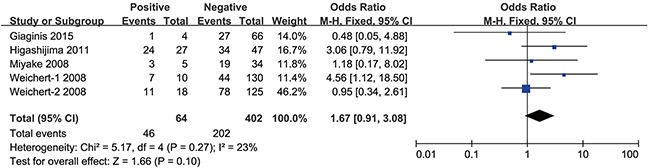
Forest plot of odds ratio (OR) Association between HDAC1 expression and distant metastasis in overall gastrointestinal malignancy.

However, the groups positive and negative for lymph node metastasis (OR = 1.49, 95% CI = 0.85–2.61, P = 0.16) (Figure 6), as well as distant metastasis (OR = 1.67, 95% CI = 0.91–3.08, P = 0.10) (Figure 7), did not show a significant difference in HDAC1 expression in the gastrointestinal cancer patients overall. In the sub-group analyses (Table 3), we found that the HDAC1 expression level was higher in the lymph node metastasis-positive group than in the negative group with gastric cancer (OR = 1.60, 95% CI = 1.07–2.40), and the group positive for distant metastasis showed higher HDAC1 expression than the negative group in patients with colorectal cancer (OR = 3.67, 95% CI = 1.38–9.79). The other analyses showed no significant difference or were short studies (no study or only one). Collectively, these findings suggest that there are tight correlations between HDAC1 expression and the clinical features of gastrointestinal cancer patients.

### Impact of HDAC1 expression on overall survival of gastrointestinal cancer patients

**Figure 8:**
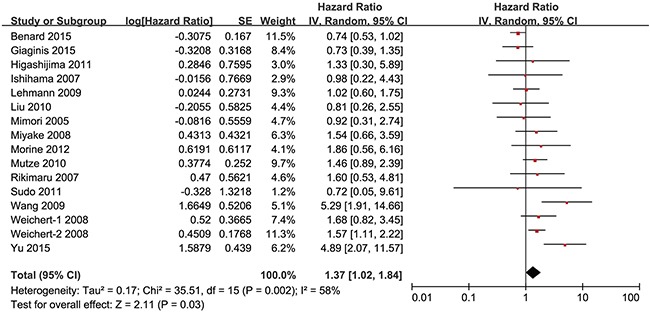
Forest plot of hazard ratio (HR) Association between HDAC1 expression and the OS of overall gastrointestinal cancer patients.

**Figure 9:**
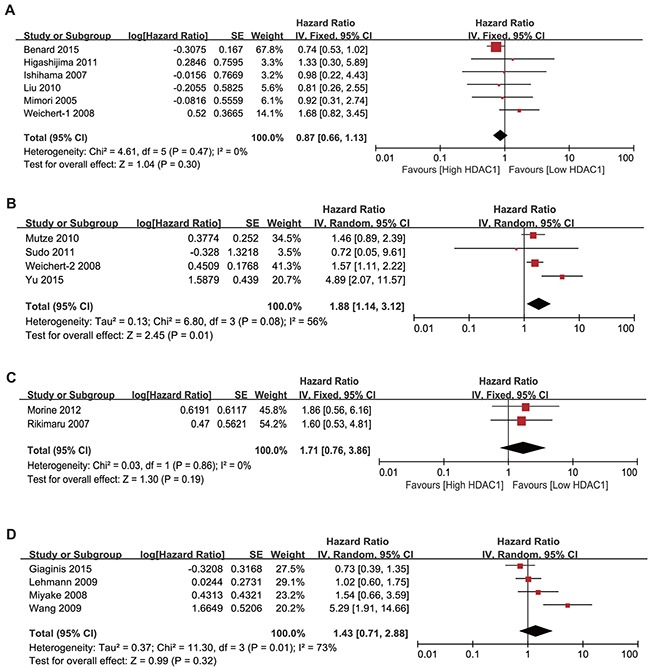
Forest plot of hazard ratio (HR) Association between HDAC1 expression and the OS of colorectal cancer **(A)**, gastric cancer **(B)**, liver cancer **(C)** and pancreatic cancer **(D)** patients.

Subsequently, we investigated the association between HDAC1 expression and the overall survival of gastrointestinal cancer patients. At first, we found that gastrointestinal cancer patients with low HDAC1 expression showed better overall survival than patients with high HDAC1 expression. The pooled hazard ratio (HR) with 95% CI is shown in Figure 8 (HR = 1.37, 95% CI = 1.02–1.84, P = 0.03). To determine whether HDAC1 expression was correlated with colorectal, gastric, pancreatic, esophageal or liver cancer prognosis, sub-group analyses were performed. We found that HDAC1 expression was negatively correlated with the OS rate of gastric cancer patients (HR = 1.88, 95% CI = 1.14–3.12, P = 0.01) (Figure 9B). However, the OS rate was comparable between patients with low HDAC1 expression and those with high HDAC1 expression in colorectal cancer (HR = 0.87, 95% CI = 0.66–1.13, P = 0.30) (Figure 9A), liver cancer (HR = 1.71, 95% CI = 0.76–3.86, P = 0.19) (Figure 9C) and pancreatic cancer (HR = 1.43, 95% CI = 0.71–2.88, P = 0.32) (Figure 9D). No study analyzed the overall survival of esophageal cancer patients. Therefore, these findings indicate that patients with low HDAC1 expression show better overall survival than those with high HDAC1 expression in gastrointestinal malignancies, especially gastric cancer.

### Sensitivity analysis and publication bias

Sensitivity analyses were performed by omitting a study at a time. The results were not significantly changed, indicating the stability of the present analyses. The funnel plots were almost symmetric, suggesting that there were no significant publication biases in these meta-analyses (Supplementary Figures 1-6).

## DISCUSSION

In our study, a combined analysis of 28 eligible clinical studies revealed a critical role of HDAC1 expression in gastrointestinal cancer progression and prognosis. The meta-analysis results suggested that the expression of HDAC1 in gastrointestinal cancer tissues, especially colorectal cancer tissue, is higher than that in noncancerous tissues. In addition, we performed meta-analyses to determine the relationship between HDAC1 expression and the clinical features of gastrointestinal malignancies, and correlations between HDAC1 expression and tumor stage, grade, lymph node metastasis and distant metastasis were observed. Finally, we found that gastrointestinal cancer patients with low HDAC1 expression showed better OS than those with high HDAC1 expression, especially with gastric cancer.

HDAC1 expression in gastrointestinal malignancies is controversial. For example, in several studies HDAC1 overexpression was detected in over 54% of gastric cancer tissues in both mRNA and protein levels [16, 17, 37], but another study [38] found that HDAC1 was downregulated in gastric tumors compared with the level in adjacent non-tumors. Additionally, the HDAC1 expression level has been reported to be increased in colorectal cancer tissue as compared to normal tissue [21], but there was no significant difference between colorectal cancer and normal tissue in another study [11]. In this analysis, we found that HDAC1 expression in colorectal cancer tissues is higher than that in normal tissues. Much more work needs to be done for other types of gastrointestinal cancer.

In this analysis, correlations between HDAC1 expression and tumor stage, tumor grade, lymph node metastasis or distant metastasis were observed in gastrointestinal malignancies, indicating that HDAC1 might be a good biomarker to distinguish different stages, grades, and states of lymph node metastasis or distant metastasis and would be beneficial for the diagnosis of gastrointestinal malignancy. Although there was no correlation between HDAC1 expression and OS of colorectal cancer, liver cancer and pancreatic cancer patients, we found that gastric cancer patients with low HDAC1 expression showed better OS than those with high HDAC1 expression, indicating that HDAC1 might be a good prognostic marker for gastric cancer patients and could help screen out high-risk patients with gastric cancer.

This study has several limitations. First, the cut-off value to determine positive or negative expression of HDAC1 varied across the included studies. Second, the number of cohorts included for some analyses was insufficient, making the results less convincing. Therefore, the role of HDAC1 expression in gastrointestinal cancer progression and prognosis warrants further study.

HDAC1 has been served as a target for cancer therapy, and small molecule HDAC inhibitors have been used in clinical treatment of patients with several types of cancer, such as T-cell lymphoma and multiple myeloma currently. Consistent with this, the present analysis revealed the important clinical value of HDAC1 expression in gastrointestinal malignancy. In conclusion, HDAC1 expression might be a good biomarker for the diagnosis and prognosis of gastrointestinal cancer patients.

## MATERIALS AND METHODS

### Search strategy and eligibility criteria

A systematic literature search was conducted for original articles analyzing the correlation between HDAC1 expression and the progression and prognosis of several types of gastrointestinal malignancy in PubMed, Embase and Web of Science. Studies were selected using the following keywords: “HDAC1” or “Histone Deacetylase 1” for HDAC1; “CRC”, “colorectal cancer” or “colorectal carcinoma” for colorectal cancer; “gastric cancer”, “gastric neoplasm”, “stomach cancer” or “stomach neoplasm” for gastric cancer; “esophagus cancer” or “esophageal neoplasm” for esophageal cancer; “hepatocellular cancer”, “hepatocellular carcinoma” or “liver cancer” for liver cancer; and “pancreatic cancer” or “pancreatic neoplasm” for pancreatic cancer. The search ended on November 20th, 2016, and no lower date limit was used. No language restriction was applied, and the references of the relevant studies were also screened to check for potentially relevant articles.

The full text of each relevant study was carefully evaluated. The studies collected in the present meta-analysis were required to meet the following criteria: (1) HDAC1 expression was measured by PCR or immunohistochemistry; (2) the clinical features or prognosis of gastrointestinal malignancy were investigated; and (3) the correlation of HDAC1 expression with clinical features and survival outcomes was analyzed. When several studies collected data from the same patient group, the most recent study was used; if the most recent study did not meet the inclusion criteria, the highest quality study was used. Articles were excluded if (1) they were case reports, letters, or reviews without original data; (2) they focused on animal models or cancer cells; (3) the expression of HDAC1 was determined by western blot; or (4) the full text was unavailable. All evaluations were independently performed by two authors, Lin-Lin Cao and Zhihong Yue, to ensure the accurate inclusion of studies. Disagreement between the two authors was resolved by reaching a consensus in accordance with the original article.

### Data extraction

Two authors, Lin-Lin Cao and Zhihong Yue, independently extracted data from the eligible studies. The following information was extracted from each included study: first author's name, publication year, country, sample size, age, HDAC1 detection method, the cut-off value, histology, stage, follow-up period and outcome. If there were no original data for HDAC1 expression and only a histogram was provided, we used Engauge Digitizer 4.1 (http://digitizer.sourceforge.net) to extract the expression data. If the cut-off value of HDAC1 expression was not provided, the mean value of all samples was considered to be the cut-off value. In addition, if hazard ratios (HRs) for overall survival (OS) rates according to HDAC1 expression were not reported directly, the number of deaths and total samples in each article were extracted for HR calculation. If only the Kaplan–Meier curves were available, the survival data were extracted using Engauge Digitizer 4.1 and analyzed as described previously [39].

### Quality score assessment

Two reviewers (Lin-Lin Cao and Zhihong Yue) independently assessed the quality of the included studies according to the Newcastle-Ottawa Scale (NOS) (http://www.ohri.ca/programs/clinical_epidemiology/oxford.asp). The scale consists of three components related to sample selection, comparability and ascertainment of exposure.

### Statistical analysis

Analysis was conducted using Review Manager 5.3 (Cochrane Collaboration, Oxford, UK). The ORs with 95% CIs were used to compare the HDAC1 expression level of cancer tissues with that of noncancerous tissues and to analyze the association between the expression level of HDAC1 and clinical features of gastrointestinal malignancy. HRs with 95% CIs were used to evaluate the correlation of HDAC1 expression with the overall survival of gastrointestinal cancer patients. The random-effect model was applied when I^2^> 50%, and the fixed-effect model was used in the absence of between-study heterogeneity (I^2^ ≤ 50%). P values of <0.05 were considered to be statistically significant. Publication bias was evaluated using the funnel plot.

## SUPPLEMENTARY FIGURES


