# Supplementary material for: The expression of histone deacetylase HDAC1 correlates with the progression and prognosis of gastrointestinal malignancy
Source: Oncotarget. 2017 Apr 5;8(24):39241–53. doi: 10.18632/oncotarget.16843 (PMC5503610; doi:10.18632/oncotarget.16843)
Supplement: Supplementary file 1 [file oncotarget-08-39241-s001.pdf]

The expression of histone deacetylase HDAC1 correlates with the progression and prognosis of gastrointestinal malignancy

SUPPLEMENTARY MATERIALS

SUPPLEMENTARY FIGURES

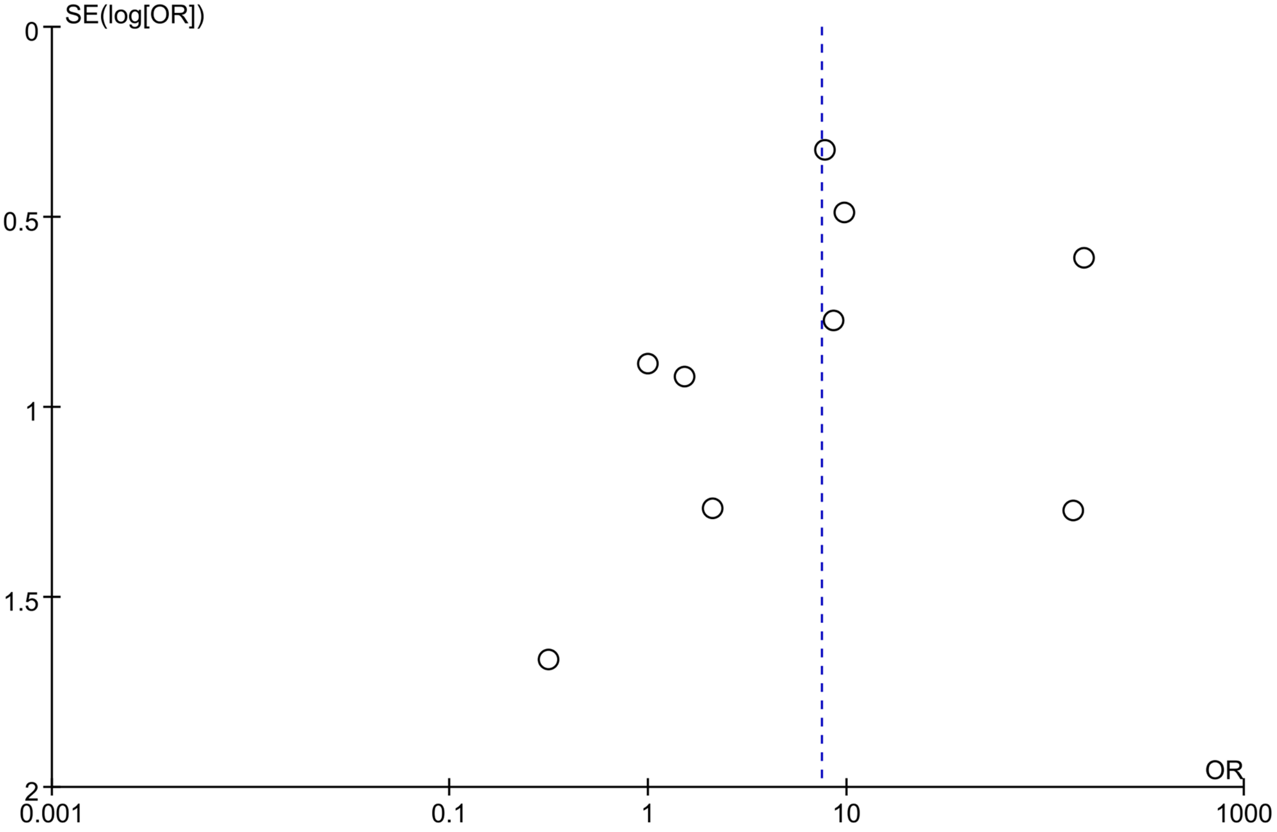

Supplementary Figure 1: Funnel plots of publication bias in the meta-analysis as shown in Figure 2.

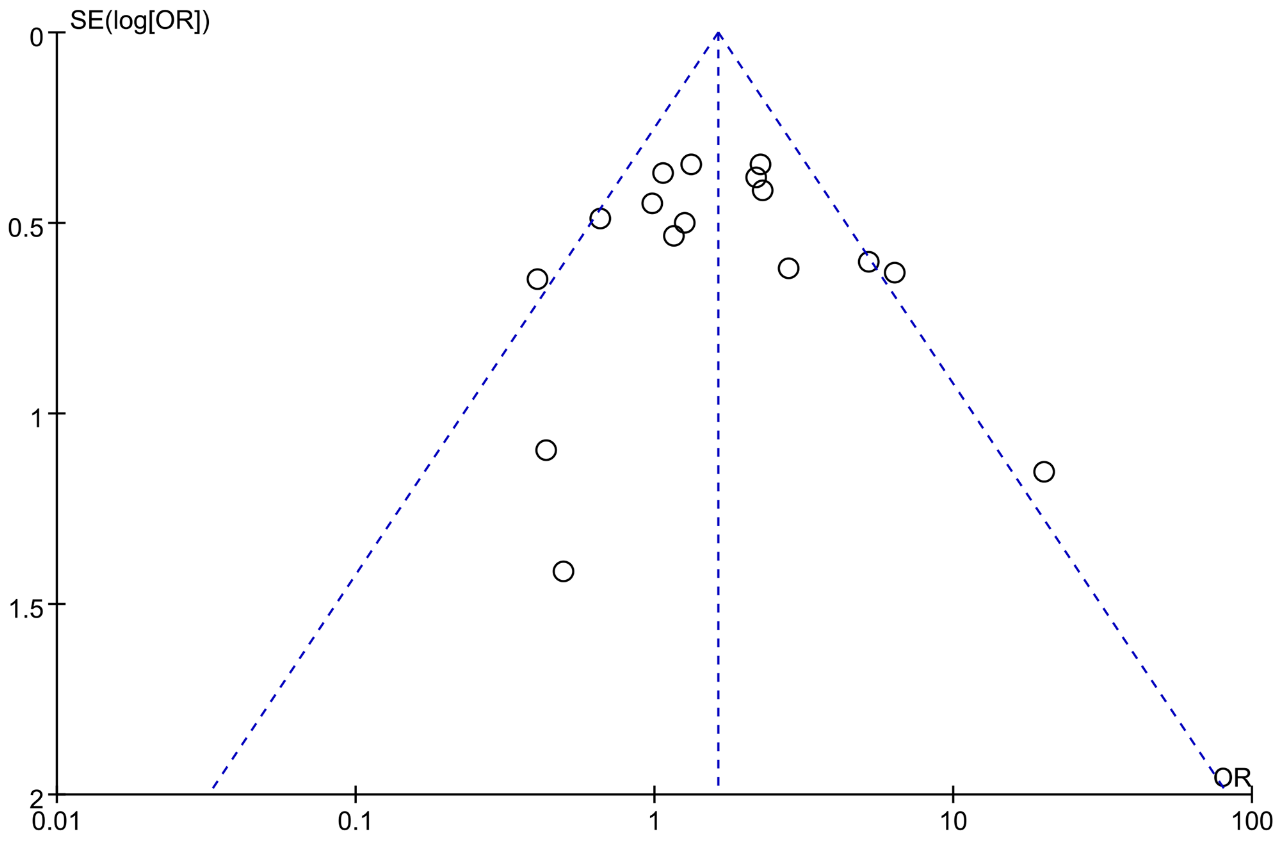

Supplementary Figure 2: Funnel plots of publication bias in the meta-analysis of HDAC1 expression and tumor stage.

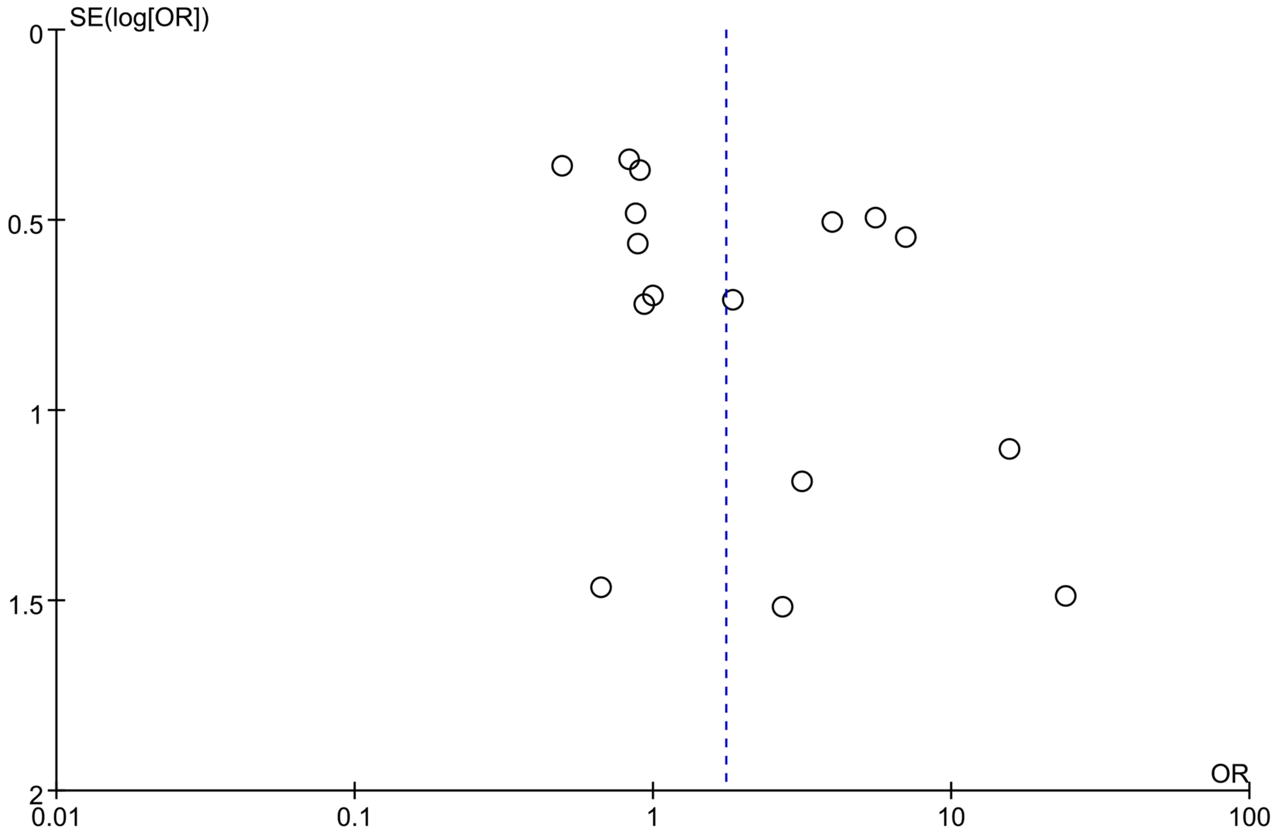

Supplementary Figure 3: Funnel plots of publication bias in the meta-analysis of HDAC1 expression and tumor grade.

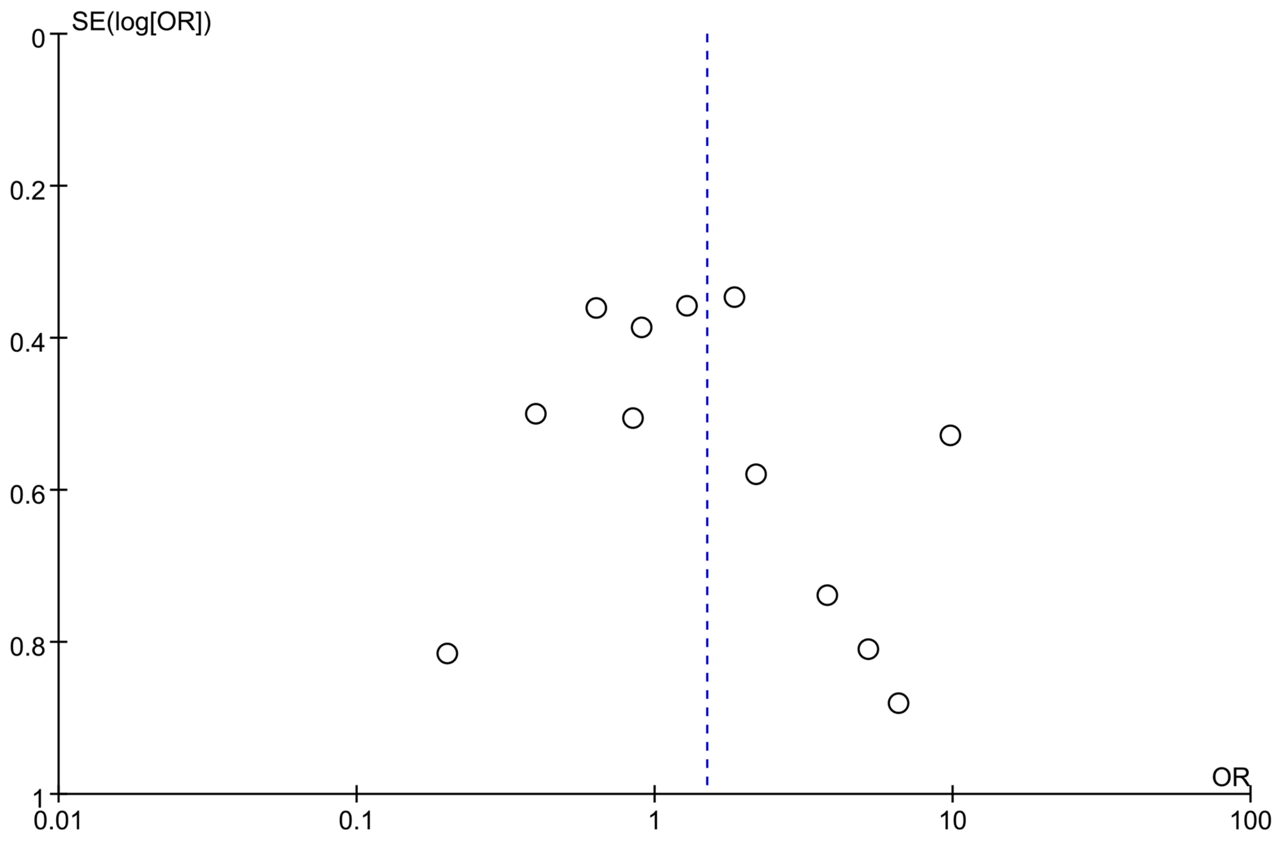

Supplementary Figure 4: Funnel plots of publication bias in the meta-analysis of HDAC1 expression and lymph node metastasis.

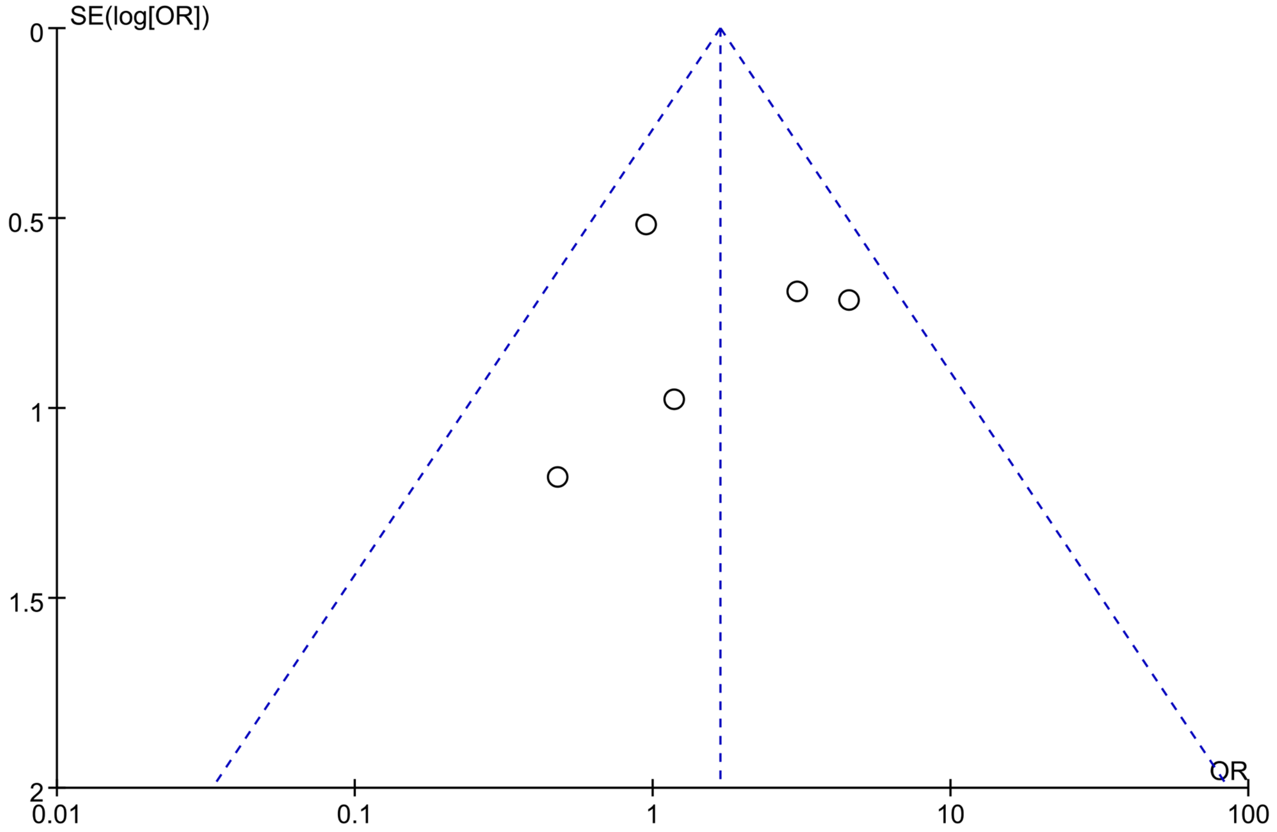

Supplementary Figure 5: Funnel plots of publication bias in the meta-analysis of HDAC1 expression and distant metastasis.

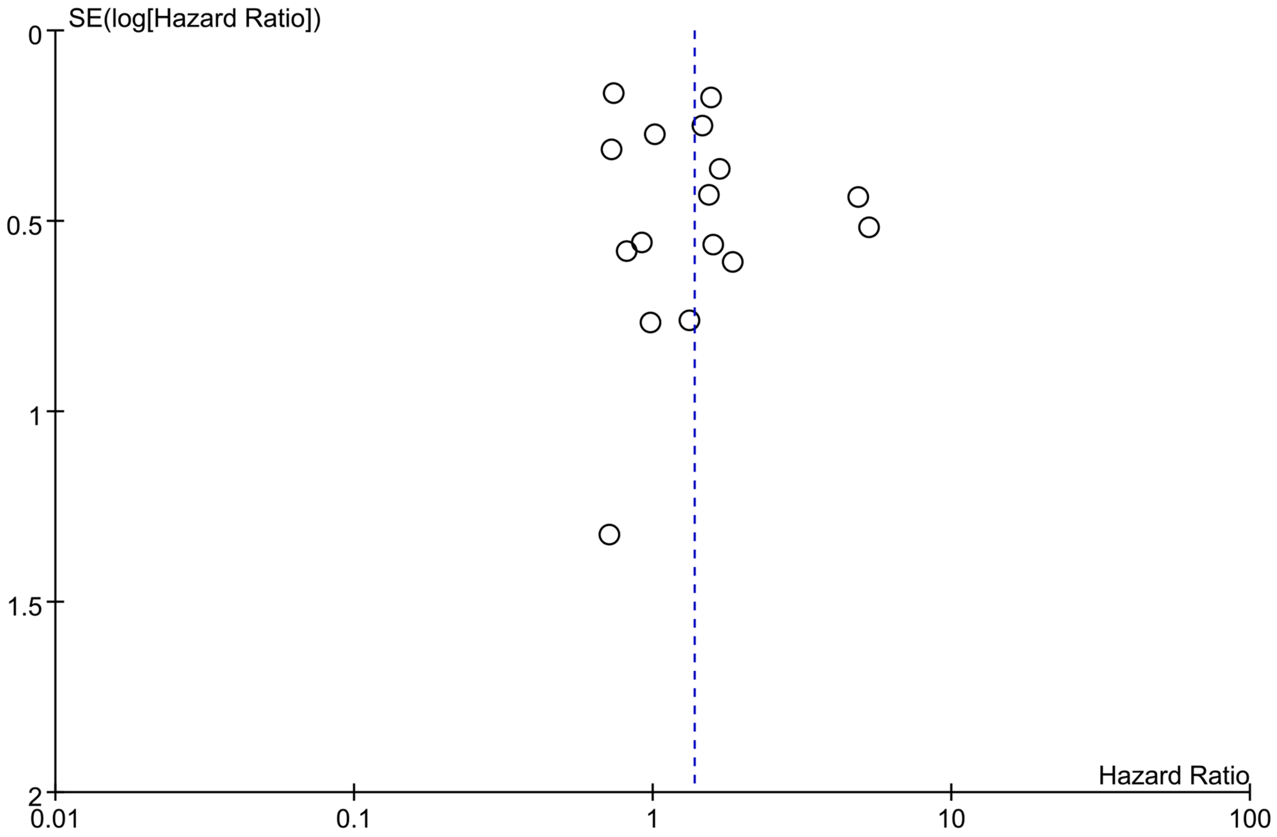

Supplementary Figure 6: Funnel plots of publication bias in the meta-analysis of HDAC1 expression and OS as shown in Figure 8.
